# Which Circulating Antioxidant Vitamins Are Confounded by Socioeconomic Deprivation? The MIDSPAN Family Study

**DOI:** 10.1371/journal.pone.0011312

**Published:** 2010-06-25

**Authors:** Dinesh Talwar, Alex McConnachie, Paul Welsh, Mark Upton, Denis O'Reilly, George Davey Smith, Graham Watt, Naveed Sattar

**Affiliations:** 1 Department of Clinical Biochemistry, Glasgow Royal Infirmary, Glasgow, United Kingdom; 2 Robertson Centre for Biostatistics, University of Glasgow, Glasgow, United Kingdom; 3 Division of Cardiovascular and Medical Sciences, University of Glasgow, Glasgow, United Kingdom; 4 Woodlands Family Medical Centre, Stockton-on-Tees, United Kingdom; 5 Medical Research Council Centre for Causal Analyses in Translational Epidemiology, University of Bristol, Bristol, United Kingdom; 6 General Practice and Primary Care, University of Glasgow, Glasgow, United Kingdom; University of Michigan, Canada

## Abstract

**Background:**

Antioxidant vitamins are often described as having “independent” associations with risk of cancer, cardiovascular disease (CVD) and mortality. We aimed to compare to what extent a range of antioxidant vitamins and carotenoids are associated with adulthood and childhood markers of socioeconomic deprivation and to adverse lifestyle factors.

**Methods and Findings:**

Socioeconomic and lifestyle measures were available in 1040 men and 1298 women from the MIDSPAN Family Study (30–59 years at baseline) together with circulating levels of vitamins A, C, E, and carotenoids (α-carotene, β-carotene, lutein and lycopene). Markers of socioeconomic deprivation in adulthood were consistently as strongly associated with lower vitamin C and carotenoid levels as markers of adverse lifestyle; the inverse association with overcrowding was particularly consistent (vitamin C and carotenoids range from 19.1% [95% CI 30.3–6.0] to 38.8% [49.9–25.3] lower among those in overcrowded residencies). These associations were consistent after adjusting for month, classical CVD risk factors, body mass index, physical activity, vitamin supplements, dietary fat and fibre intake. Similar, but weaker, associations were seen for childhood markers of deprivation. The association of vitamin A or E were strikingly different; several adult adverse lifestyle factors associated with *higher* levels of vitamin A and E, including high alcohol intake for vitamin A (9.5% [5.7–13.5]) and waist hip ratio for vitamin E (9.5% [4.8–14.4]), with the latter associations partially explained by classical risk factors, particularly cholesterol levels.

**Conclusions:**

Plasma vitamin C and carotenoids have strong inverse associations with adulthood markers of social deprivation, whereas vitamin A and E appear positively related to specific adverse lifestyle factors. These findings should help researchers better contextualize blood antioxidant vitamin levels by illustrating the potential limitations associated with making causal inferences without consideration of social deprivation.

## Introduction

Low circulating levels of “antioxidant” vitamins, such as vitamin C, are associated with increased risk of atherothrombotic events in prospective studies of general populations [1]. Speculatively, this has been mechanistically linked to a putative anti-atherogeneic effect of these vitamins, perhaps by lowering local concentrations of reactive oxygen intermediates, and in turn lessening low density lipoprotein oxidation and attenuating endothelial dysfunction. Low plasma levels of antioxidant vitamins appear also to be associated with higher risk of cancer incidence and mortality as well as with CVD [1]–[5] and the non-specificity of such associations perhaps argues against a causal role [6]. Randomised controlled trials of supplements of antioxidant vitamins have thus far failed to show any consistent efficacy in reducing vascular risk or cancer [7]–[13]. This failure to reduce risk is unlikely to be only due to insufficient study length [8], [12]. The inconsistent findings from observational studies on the one hand, and trial evidence on the other, require further interrogation. A better understanding of the determinants of vitamin levels in populations would have several advantages, not least to re-focus on the “upstream” causes of vascular risk.

Lawlor et al have previously shown that adult and childhood markers of socioeconomic deprivation are related to circulating levels of vitamins C and E in the British Women's Heart and Health Study [14]. Moreover, after adjusting for multiple factors relating to adverse lifestyle and deprivation, vitamin C is no longer associated with risk of coronary heart disease [15]. Social deprivation is of current research interest as an informative factor in CVD risk prediction in the UK [16], [17]. The association of a wide range of antioxidant vitamins with a range of socioeconomic factors across the lifecourse requires exploration in order to assess: whether associations of various antioxidant vitamins with such factors are uniform, which socioeconomic/lifestyle markers are most strongly associated with vitamins, and to directly compare the impact of socioeconomic versus lifestyle risk factors on vitamin levels.

Using the well-phenotyped MIDSPAN Family Study (MFS) [18], with data on socioeconomic and lifestyle markers over two generations, we aimed to comprehensively explore the associations of a wide range of antioxidant vitamins and carotenoids (vitamins A,C,E, α-carotene, β-carotene, lutein and lycopene) with various measures of socioeconomic position and markers of adverse lifestyle both in adulthood and childhood.

## Methods

### Recruitment

The MFS took place between March and December 1996. MFS included adult sons and daughters of couples who had both participated in the Renfrew/Paisley prospective cohort study between 1972 and 1976, which consisted of 7049 men and 8353 women aged between 45 and 64, who lived in the towns of Renfrew and Paisley, and included 4064 known married couples [18]. Renfrew and Paisley are two similar towns situated on the south-west perimeter of the City of Glasgow within a large post-industrial area. In 1993–94, the couples (or if both were deceased, the informant on the death certificate) were written to for information on any offspring. Offspring aged 30–59 years and living locally (in 1996) formed the eligible population (3202 offspring from 1767 families). In all, 1040 male and 1298 female offspring from 1477 families took part, an individual response rate of 73% and a family response rate of 84%. Ineligible offspring (n = 1813) included those too old or too young, those not living locally, those with problematic addresses (addresses provided by parents or death certificate informants which, for reasons of completeness or accuracy, could not be located in a current postcode directory) or who died before the study commenced. The offspring were invited, in random order, to attend a screening examination, similar to their parents 20 years previously. All information on physical activity, smoking, occupation, diet, and alcohol consumption were based on self-reported answers from standard questionnaires. The total units of alcohol consumed per week was obtained from questions on beer, spirits and wine (and similar drinks) consumption each day of the previous week [20]. Participants were asked to report their daily activity as: very physically active, fairly physically active, not very physically active, or not at all physically active. They also reported how often per week they were physically active in their nonworking time: more than three times, two to three times, once, less than once, and never. Participants who were not very physically active or not at all physically active during their usual daily activities and who were physically active outside work less than once a week or never, were classified as being sedentary [19]. Nutrient intake was estimated using an established food frequency questionnaire [19]. Nutrient (including fat and fibre) intakes were calculated by a computer program, which multiplied the food frequency by standard portion size, and by nutrient values from UK food composition tables [19]. Measurements of height, weight, waist, hip and blood samples were obtained by a qualified research nurse who also collected non-fasting venous blood samples. Blood samples obtained were spun down, plasma separated, aliquoted, and stored at −80 C for subsequent analysis. Ethical approval for the study was obtained from the Argyll and Clyde Health Board Local Research Ethics Committee on 17 July 1995, and from Greater Glasgow Health Board Local Research Ethics Committee on 11 May 1995. Informed written consent was obtained from all participants for venepuncture, sample storage and record linkage.

### Socioeconomic generalisability of the cohort

The original MIDSPAN cohort was typical of the area in being characterised by high levels of socioeconomic deprivation and high levels of premature mortality, which are more severe than Scotland (and England) as a whole but less severe than the poorest areas of Glasgow. The second generation (MFS) is socially mobile [21]. The MFS had virtually the same social class distributions in childhood and in adulthood as participants of the nationally representative 1958 birth cohort study [21].

### Markers of deprivation and lifestyle

The socioeconomic and lifestyle circumstance of the participants in adulthood were derived from self-reported questionnaire responses and physical examination measures. For simplicity, these were primarily used as dichotomous variables, although secondary analyses, using variables in a continuous fashion are also reported. Socioeconomic factors were: occupational social class coded according to the registrar general's classification (manual vs. non-manual); Carstairs deprivation index (“DepCat”: an index of deprivation in a specific postcode based on census data comprising information on low social class, lack of car ownership, overcrowding and male unemployment; a score of 1 is most affluent, a score of 7 is most deprived) [22] (continuous scores and categories 5–7 vs. 1–4 were used); highest educational level (secondary or lower vs. tertiary); housing tenure (rented vs. owner occupier); domestic overcrowding (number of people sharing accommodation ≥ number of available rooms vs. otherwise); access to cars (none vs. one or more). Lifestyle factors were: high alcohol intake (>28 [men] or 21 [women] units per week vs. otherwise); low fibre intake (<18 g/day vs. ≥18 g/day); high fat intake [>35% of total food energy intake vs. ≤35%]; low physical activity [not very/not at all physically active during usual daily activities, and physically active for at least 20 minutes on less than one occasion a week during non-working time, vs. otherwise]; current smoking habit (smoker vs. non-smoker); two measures of obesity (body mass index (BMI) >30 kg/m^2^ vs. ≤30 kg/m^2^; waist-hip ratio (WHR) >1.0 [men] or 0.9 [women] vs. otherwise). Participant's log-transformed cholesterol levels were expressed as continuous variables only.

Socioeconomic and lifestyle factors during childhood were inferred from the questionnaire and examination results of their parents, when surveyed 20 years previously, from the participants' questionnaire responses relating to their childhood circumstances, or from adult physiological measures indicative of poor childhood nutrition. Those factors with equivalent adult measures were dichotomized in the same way. Socioeconomic factors were: occupational social class and Carstairs deprivation index (from parental questionnaire responses); housing tenure, domestic overcrowding and car access (from MFS participants' questionnaire responses about their childhood). Lifestyle factors were: maternal/paternal smoking status (from parental questionnaire responses) and BMI (from parental examination); leg length (from MFS participants' physical examination, categorised as < vs. ≥ the sex-specific median value). Maternal and paternal heights were expressed as continuous variables only.

### Laboratory Methods

Plasma vitamin A (retinol), vitamin E (α-tocopherol) and the carotenoids lutein, lycopene, α-carotene and β-carotene concentrations were determined by a high-performance liquid chromatography (HPLC) method. Briefly, plasma was deproteinised with ethanol containing internal standards and extraction of the analytes of interest was performed using hexane. Analysis was carried out using reversed-phase HPLC (5 µm microbore, Phenomenex, Macclesfield, UK) and dual wavelength monitoring (Waters, MA, USA). The limit of sensitivity for retinol and a-tocopherol was 0.3 and 3.0 µmol/l, respectively. The limit of sensitivity for lutein, lycopene, α-carotene and β-carotene was 10 µg/l. The intra-assay and inter-assay coefficient of variation was less than 9% and 11% respectively for all analytes over the sample concentration range.

Vitamin C status was assessed by measuring ascorbic acid in plasma using HPLC with electrochemical detection. The measurement in plasma was based on the method of Margolis and Davis [23]. Briefly, plasma was stabilized and deproteinized with an equal volume of 60 g/L metaphosphoric acid (MPP) within 4 hrs of collection. After centrifugation, one part of the supernatant was diluted with four parts of a freshly prepared solution of MPP (30 g/L) and Dithiothreitol (DTT; 1.5 g/L) and 25 ul injected onto a reverse phase HPLC column (5-um, Apex ODS; 4.6×250 mm) via an autosampler. Isocratic elution was used at a flow rate of 1.5 mL/min. with a mobile phase consisting of 14.2 g of chloroacetic acid, 4.6 g of sodium hydroxide, 0.86 g of sodium EDTA and 200 mg of octane sulphonic acid (sodium salt) in 1 L distilled water. The electrochemical detector (ESA Coulochem 5100) was set at +0.4 V and the ascorbic acid quantified by peak-height measurements and electronic peak integration. The limit of sensitivity of the method is 0.5 µmol/L and the intra and inter-assay CV less than 2% and 4% respectively.

Cholesterol was measured by routine methods on a non-fasting serum sample [19].

### Statistical Methods

We summarised the variables under study for the full dataset and for those with complete data only, to assess whether the subgroup with complete data were in any way different to the rest of the population. We compared males and females, using t-tests of log-transformed vitamin levels, or χ^2^-tests for binary indicators of socioeconomic or lifestyle factors. Spearman correlation coefficients were used to assess associations between deprivation markers as binary variables.

For each vitamin, we constructed a series of linear regression models and then multivariable logistic regression models to determine the associations with each measure of socioeconomic deprivation or adverse lifestyle factor. Vitamin variables (and relevant covariables) were log-transformed prior to analysis when they were non-normally distributed. Each model included age as a linear term, sex as a binary term, and calendar month as a categorical term, to allow for seasonal variations. The effect estimate and 95% confidence interval for the socioeconomic or lifestyle marker was then extracted; the effect estimates for all markers are reported graphically and in tabular form with effects back-transformed and expressed as the percentage relative difference.

## Results

### Available data

Of the 2338 participants, vitamin C was measured in 87.1%, vitamins A and E in 92.9%, and α-carotene, β-carotene, lutein, and lycopene in 92.6%. A complete data set was available in 79.4% of the population. Since none of the results were different when analysed on those with complete data only, each analysis reported here includes all subjects with available data.

### Baseline Characteristics

Baseline characteristics of the population are summarised in **Table 1**. Age was similar in male and female participants. Vitamin C, α-carotene and β-carotene had lower levels in males than in females (p<0.001). Levels of vitamin A, vitamin E and lycopene were higher in males (p<0.009). Levels of lutein were not found to differ between males and females (p = 0.10).

**Table 1:** Baseline characteristics of study population.

	Males		Females		p-value
	All	Complete	All	Complete	
	n≤1040	N = 838	n≤1298	N = 1019	
Age (years)	44.9 (6.3)	45.0 (6.2)	45.2 (6.1)	45.4 (6.1)	p = 0.16
Vitamin C ( µmol/l)	35.7 (2.0)	34.9 (2.0)	45.4 (1.8)	45.2 (1.8)	p<0.001
Vitamin A ( µmol/l)	2.3 (1.3)	2.3 (1.3)	1.9 (1.3)	1.9 (1.3)	p<0.001
Vitamin E ( µmol/l)	33.9 (1.4)	34.2 (1.4)	32.2 (1.3)	32.1 (1.3)	p<0.001
Lutein ( µg/dl)	13.1 (1.6)	13.0 (1.6)	13.5 (1.6)	13.3 (1.5)	p = 0.10
Lycopene ( µg/dl)	17.7 (1.9)	17.8 (1.9)	16.5 (1.8)	16.5 (1.8)	p = 0.009
α-Carotene ( µmol/l)	2.7 (2.5)	2.6 (2.5)	3.8 (2.3)	3.7 (2.3)	p<0.001
β-Carotene ( µmol/l)	12.6 (2.3)	12.6 (2.3)	18.9 (2.0)	19.1 (2.0)	p<0.001

Summaries shown are geometric mean and exponential of standard deviation (SD) on a logarithmic scale, except for age, which is summarised as mean (SD). ‘Complete’ refers to the subset of individuals with complete data on all vitamin, socioeconomic and lifestyle factors. p-values are from two-sample t-tests between males and females on a logarithmic scale, except for age, for which a two-sample t-tests was applied on the original scale.

Of the adult socioeconomic and lifestyle factors (**Table 2**) men were more likely to be defined as being manual social class, have a high alcohol intake, and have a raised waist-hip ratio (p<0.009). Females were more likely to lack a tertiary education, have no access to a car, and to have a high fat diet (p≤0.02). When analysed as a continuous variable, BMI was slightly higher among men (p<0.001), although less food energy was obtained from fat (p<0.01).

**Table 2:** Adult socioeconomic and lifestyle factors for study population.

	Males		Females		p-value
	All	Complete	All	Complete	
	n≤1040	n = 838	n≤1298	n = 1019	
**Socioeconomic factors**					
Manual social class	432 (41.5%)	347 (41.4%)	298 (23.0%)	244 (23.9%)	p<0.001
DepCat (category5-7)	340 (32.7%)	272 (32.5%)	430 (33.2%)	342 (33.6%)	p = 0.83
No tertiary education	387 (37.3%)	312 (37.2%)	738 (57.0%)	588 (57.7%)	p<0.001
Not owner-occupier	153 (14.7%)	120 (14.3%)	211 (16.3%)	171 (16.8%)	p = 0.33
Overcrowding	33 (3.2%)	25 (3.0%)	39 (3.0%)	30 (2.9%)	p = 0.91
No car access	124 (11.9%)	90 (10.7%)	201 (15.5%)	159 (15.6%)	p = 0.02
Social class (1 = I, 6 = V)*	2.94 (1.29)	2.94 (1.29)	3.01 (1.21)	3.05 (1.23)	p = 0.30
DepCat (continuous)*	3.76 (1.60)	3.75 (1.60)	3.76 (1.56)	3.78 (1.55)	p = 0.98
**Lifestyle factors**					
High alcohol intake	264 (25.4%)	226 (27.0%)	32 (2.5%)	27 (2.6%)	p<0.001
Raised waist-hip ratio	91 (8.8%)	75 (8.9%)	75 (5.9%)	56 (5.5%)	p = 0.009
Obese	186 (17.9%)	150 (17.9%)	232 (18.1%)	178 (17.5%)	p = 0.93
Low-fibre diet	254 (24.4%)	205 (24.5%)	349 (26.9%)	287 (28.2%)	p = 0.18
High-fat diet	353 (36.4%)	301 (35.9%)	617 (50.7%)	515 (50.5%)	p<0.001
Inactive	732 (70.5%)	599 (71.5%)	936 (72.2%)	738 (72.4%)	p = 0.39
Current smoker	264 (25.4%)	202 (24.1%)	326 (25.1%)	255 (25.0%)	p = 0.92
Alcohol intake (units/week)*	19.7 (19.3)	20.7 (20.1)	6.4 (6.6)	6.4 (6.6)	p<0.001
Waist-hip ratio*	0.91 (0.06)	0.91 (0.06)	0.78 (0.07)	0.78 (0.07)	p<0.001
BMI (kg/m^2^)*	26.5 (4.0)	26.6 (3.8)	25.9 (4.9)	25.8 (4.9)	p<0.001
Fibre intake (g/day)*	23.6 (7.9)	23.4 (7.5)	23.2 (7.7)	22.9 (7.5)	p = 0.19

Summaries are number (percentage) of individuals with the stated characteristic for categorical variables; p-values by χ^2^ between males and females. Continuous variables (*) values are mean (standard deviation); p-values by Wilcoxon tests.

Childhood socioeconomic markers of deprivation (**Table 3**) among males and females were similar, although females reported greater frequency of parents who were not home owners (p = 0.03). No differences were observed between men and women in parental obesity or parental smoking. Men had longer leg length than women (p<0.001), although this was adjusted for in subsequent analysis by expressing this data dichotomised at the sex-specific median.

**Table 3:** Childhood socioeconomic and lifestyle factors for study population.

	Males		Females		p-value
	All	Complete	All	Complete	
	n≤1040	n = 838	n≤1298	n = 1019	
**Socioeconomic factors**					
Father manual social class	714 (68.7%)	569 (67.9%)	902 (69.5%)	714 (70.1%)	p = 0.70
Father DepCat 5-7	611 (58.8%)	490 (58.5%)	749 (57.7%)	595 (58.4%)	p = 0.64
Parents not owner-occupiers	857 (82.6%)	696 (83.1%)	1115 (85.9%)	874 (85.8%)	p = 0.03
Childhood overcrowding	828 (79.8%)	670 (80.0%)	1058 (81.5%)	836 (82.0%)	p = 0.31
Parents no car access	496 (47.7%)	409 (48.8%)	668 (51.5%)	536 (52.6%)	p = 0.08
Father social class (1 = I, 6 = V)*	3.80 (1.24)	3.77 (1.24)	3.85 (1.24)	3.86 (1.21)	p = 0.35
Father DepCat (continuous)*	4.51 (1.34)	4.51 (1.36)	4.53 (1.27)	4.55 (1.27)	p = 0.93
**Lifestyle factors**					
Father obese	125 (12.0%)	105 (12.5%)	140 (10.8%)	110 (10.8%)	p = 0.39
Mother obese	151 (14.5%)	124 (14.8%)	204 (15.7%)	158 (15.5%)	p = 0.46
Father ever smoker	818 (78.7%)	658 (78.5%)	1028 (79.2%)	812 (79.7%)	p = 0.79
Mother ever smoker	564 (54.2%)	460 (54.9%)	702 (54.1%)	535 (52.5%)	p = 0.98
Leg length < median	517 (49.9%)	410 (48.9%)	644 (49.8%)	509 (50.0%)	p = 0.99
Father BMI (kg/m^2^)*	26.01 (3.37)	26.04 (3.42)	26.06 (3.26)	26.06 (3.23)	p = 0.73
Mother BMI (kg/m^2^)*	25.95 (4.43)	26.07 (4.53)	26.03 (4.44)	25.99 (4.44)	p = 0.43
Leg length (mm)*	816.4 (42.5)	816.6 (42.1)	742.6 (38.4)	742.6 (38.0)	p<0.001

Summaries are number (percentage) of individuals with the stated characteristic for categorical variables; p-values by χ^2^.

Markers of socioeconomic deprivation, as expected, were generally statistically associated with each other across the lifecourse (**Table 4**), although few of the associations could be described as strong among these binary variables. Interestingly, overcrowding was weakly associated with other deprivation markers in adulthood, with associations being stronger for childhood overcrowding.

**Table 4:** Spearman correlation coefficients, with p-values, between adult and childhood SES indicators (as binary measures).

	Adulthood					Childhood				
	DepCat 5–7	No tertiary education	Not owner-occupier	Overcrowding	No car access	Manual occupation	DepCat 5–7	Non owner-occupier	Overcrowding	No car access
**Adulthood**										
Manual occupation	r = 0.207*	r = 0.248*	r = 0.257*	r = 0.070†	r = 0.186*	r = 0.201*	r = 0.134*	r = 0.121*	r = 0.141*	r = 0.136*
DepCat 5–7		r = 0.146*	r = 0.271*	r = 0.052‡	r = 0.239*	r = 0.066†	r = 0.182*	r = 0.143*	r = 0.081†	r = 0.064 †
No tertiary education			r = 0.174*	r = 0.042	r = 0.219*	r = 0.200*	r = 0.112*	r = 0.163*	r = 0.161*	r = 0.165*
Non owner-occupier				r = 0.129*	r = 0.440*	r = 0.151*	r = 0.077†	r = 0.136*	r = 0.143*	r = 0.143*
Overcrowding					r = 0.062‡	r = 0.049‡	r = 0.045	r = 0.030	r = 0.071†	r = 0.071†
No car access						r = 0.126*	r = 0.057‡	r = 0.078†	r = 0.103*	r = 0.110*
**Childhood**										
Manual occupation							r = 0.148*	r = 0.287*	r = 0.290*	r = 0.251*
DepCat 5–7								r = 0.173*	r = 0.202*	r = 0.062‡
Non owner-occupier									r = 0.372*	r = 0.249*
Overcrowding										r = 0.223*

*indicates p<0.001,

†indicates p<0.01,

‡indicates p<0.05,

No symbol indicates p>0.05.

### Associations between markers of socioeconomic deprivation and adverse lifestyle with circulating levels of vitamins

#### Vitamin C associations

**Figure 1:**
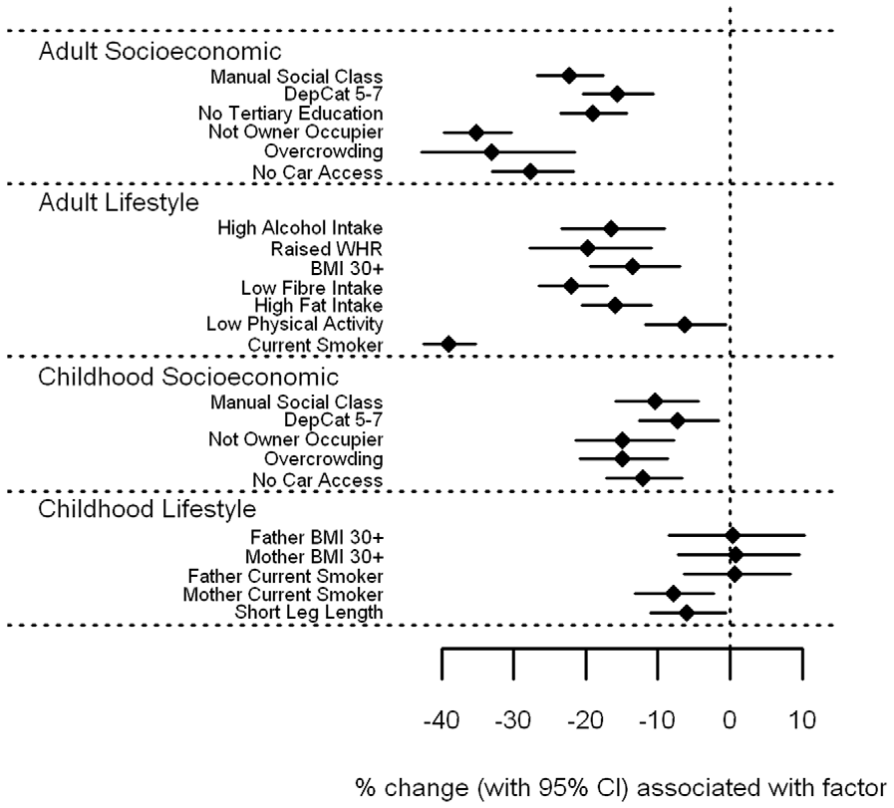
Associations between vitamin C and carotenoids and adult/childhood socioeconomic/lifestyle factors.

Vitamin C (**Figure 1** [tabulated data in **File S1**]) showed a strong inverse association with all markers of socioeconomic position and adverse lifestyle in adulthood (p<0.001) with the exception of low physical activity, where the evidence for an association was weaker (p = 0.03). A particularly strong association was found between smoking and vitamin C, with vitamin C levels in smokers estimated to be 38.9% (95% CI 35.2–42.4%) lower than non-smokers. Amongst the socioeconomic markers, measures of housing tenure and overcrowding demonstrated large effects (35.2% and 33.0% lower levels, respectively). Whilst vitamin C levels were lower among those with childhood markers of socioeconomic deprivation, the associations were less marked than for similar factors from adulthood. In general, measures of adverse lifestyle in childhood had little bearing on vitamin C: only having a mother who smoked (p = 0.007) and short leg length (p = 0.03), a marker of childhood under-nutrition, showed any association with vitamin C levels in adulthood.

#### Carotenoid associations

**Figure 2:**
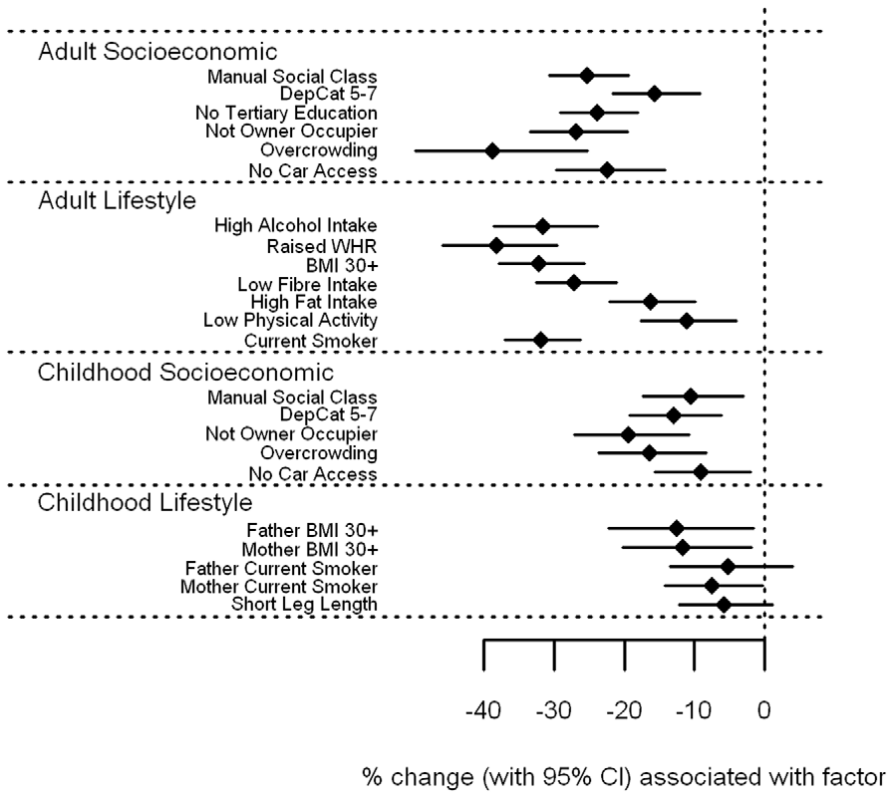
Associations between α-carotene and carotenoids and adult/childhood socioeconomic/lifestyle factors.

α-carotene, β-carotene, lutein, and to a more moderate extent lycopene (**Figures 2** **–** [Fig pone-0011312-g003] [Fig pone-0011312-g004] **5** and **File S1**) showed broadly similar inverse associations with markers of adult and childhood socioeconomic and lifestyle factors as did vitamin C. Of note, there was a marked association between high alcohol intake and low levels of β-carotene; those reporting alcohol consumption above the 28/21 units per week threshold had β-carotene levels 44.6% (38.9–49.8%) lower than those with more moderate consumption. In general, current markers of socioeconomic deprivation and adverse lifestyle were all strongly associated with low α-carotene, β-carotene, leutin, and lycopene levels in adulthood. Childhood socioeconomic circumstance generally had a lesser association with circulating levels, while little or no association was seen with adverse childhood lifestyle markers. For fat soluble α- and β-carotene, lutein and lycopene, these associations were essentially unchanged after expressing the circulating levels as a vitamin:cholesterol ratio (**File S1**).

**Figure 3 pone-0011312-g003:**
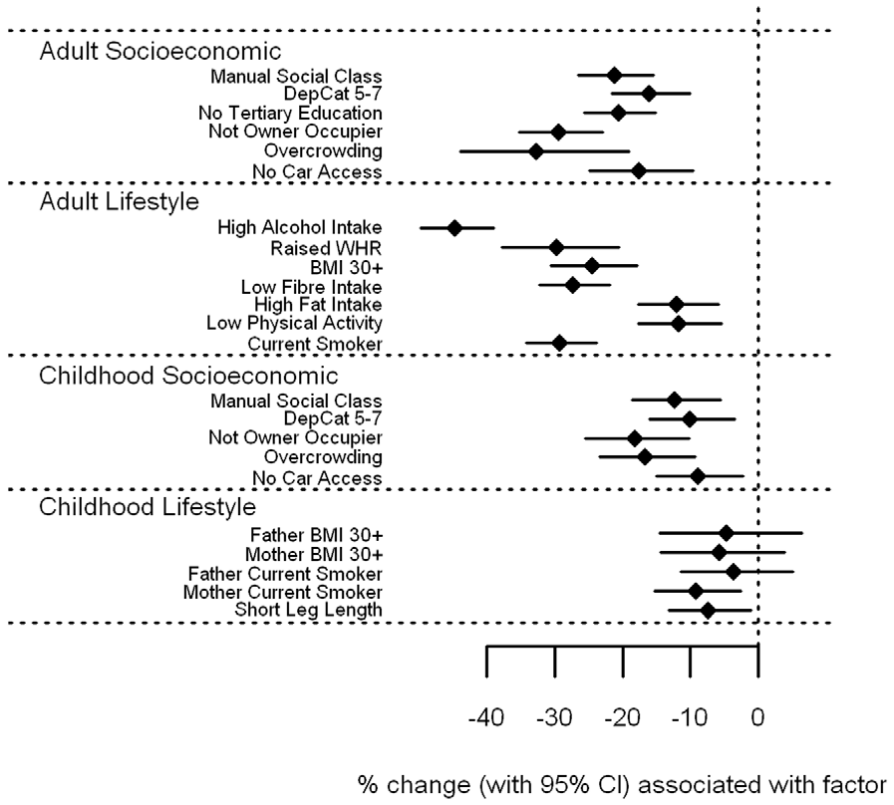
Associations between β-carotene and carotenoids and adult/childhood socioeconomic/lifestyle factors.

**Figure 4 pone-0011312-g004:**
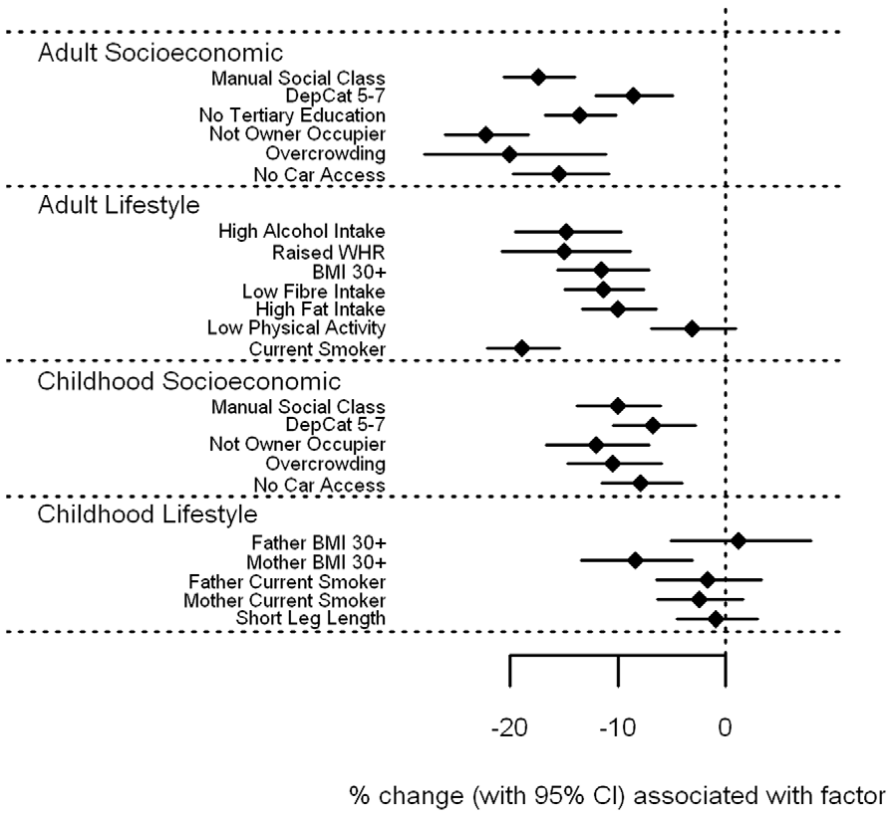
Associations between leutin and carotenoids and adult/childhood socioeconomic/lifestyle factors.

**Figure 5:**
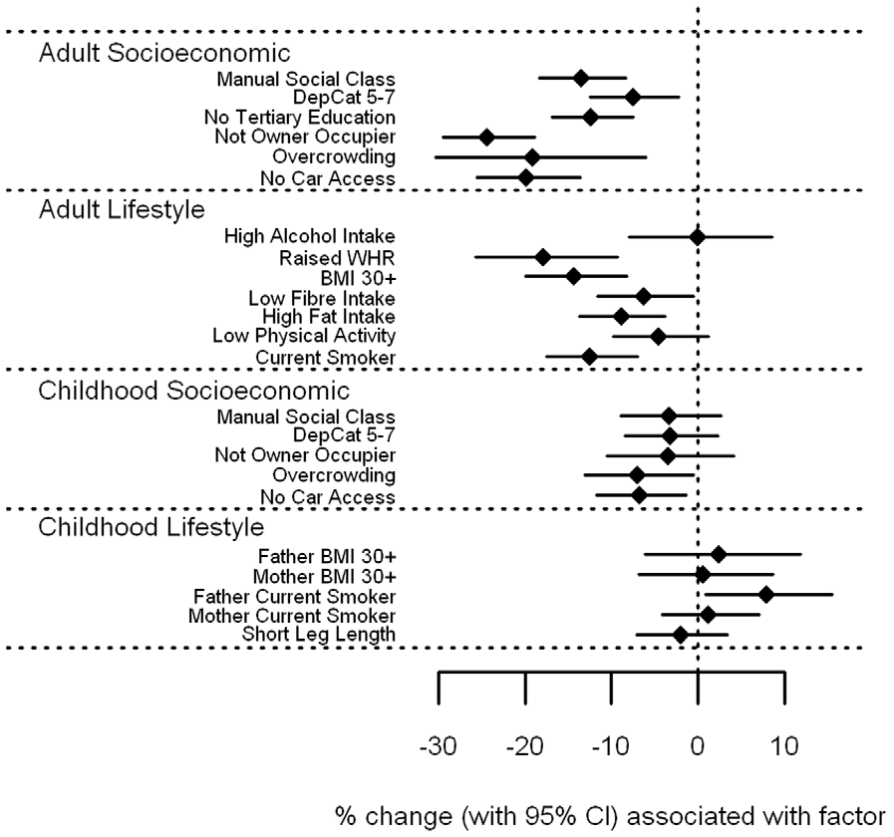
Associations between lycopene and carotenoids and adult/childhood socioeconomic/lifestyle factors.

#### Vitamin A and E associations

**Figure 6:**
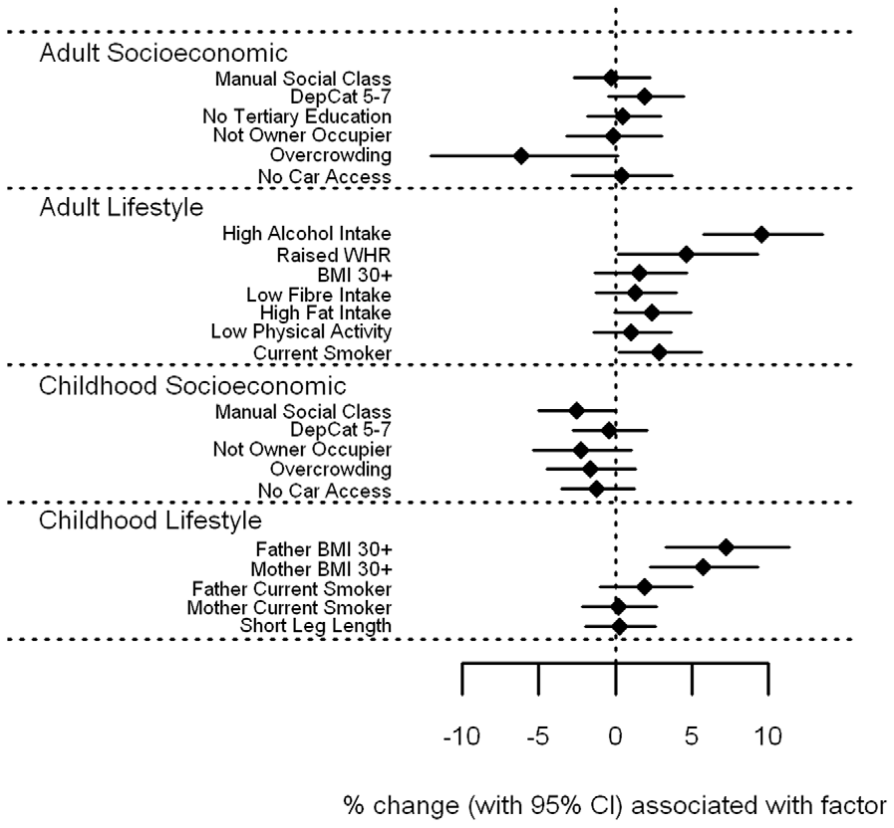
Associations between vitamin A and carotenoids and adult/childhood socioeconomic/lifestyle factors.

Associations of circulating levels of vitamin A and vitamin E with socioeconomic circumstance and adverse lifestyle were strikingly different to the other vitamins (**Figure 6** **–** **7** and **File S1**). There were few, generally weak associations between levels of vitamin A and E with socioeconomic circumstance in adulthood and childhood. Both showed weak trends for lower levels associated with currently living in overcrowded accommodation (p = 0.06 and 0.07 for vitamin A and E respectively), and with having a father who worked in a manual occupation (p = 0.05 and 0.08). In addition, vitamin E levels were 3.2% lower (0.1–6.2%) for those who were not owner-occupiers, whether measured in adulthood (p = 0.04) or in childhood (p = 0.06). In contrast, several adverse lifestyle factors were associated with *higher* levels of vitamin A and E. In particular, vitamin A levels were higher among those with high alcohol intake by an estimated 9.5% (5.7–13.5%, p<0.001), but also in those with raised WHR by 4.6% (p = 0.04), in smokers by 2.9% (p = 0.03) and those with high fat intake by 2.4% (p = 0.05). In relation to childhood lifestyle factors, an obese father was associated with 7.2% higher vitamin A levels (3.3–11.3%, p<0.001), and an obese mother with 5.7% (2.3–9.3%, p = 0.001). Vitamin E levels were raised among those who were overweight themselves; by 9.5% (4.8–14.4%) for those with a raised WHR, and by 6.6% (3.5–9.9%) for those with a BMI of 30 kg/m^2^ or more (both p<0.001), but was reduced by 2.7% in those who smoked (p = 0.04). Those with leg lengths below the sex-specific median value had 4.8% higher vitamin E levels (2.4–7.3%, p<0.001), and having an obese father was associated with an increase of 4.4% (0.4–8.5%, p = 0.03). Once fat soluble vitamin E was expressed as a vitamin:cholesterol ratio (**Figure 8** and **File S1**), the positive association with measures of obesity were largely explained, with those having a BMI of 30 kg/m^2^ or more having increased vitamin E/cholesterol ratios by 2.1%, p = 0.12, and for raised WHR, by 3.4%, p = 0.083. The association with father's obesity disappeared (p = 0.18), as did the association with short leg length (p = 0.14), although the inverse association with current smoking became stronger, with a reduction of 4.3% (2.2–6.4%), p<0.001. Similarly, previously unseen associations were found; those with a low fibre intake had lower vitamin E/cholesterol ratios by 2.9% (0.7–5.1%), p = 0.01, and those with a high fat intake had lower levels by 4.2% (2.3–6.2%), p<0.001. However, associations with socioeconomic factors, both in adulthood and in childhood, were essentially the same.

**Figure 7:**
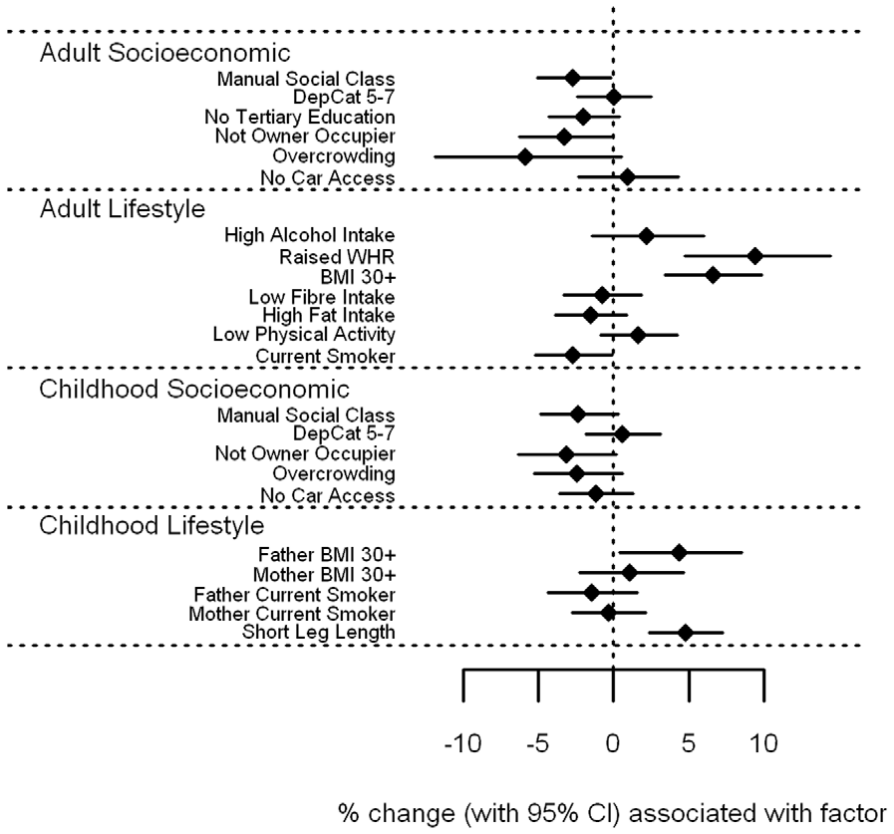
Associations between vitamin E and carotenoids and adult/childhood socioeconomic/lifestyle factors.

**Figure 8:**
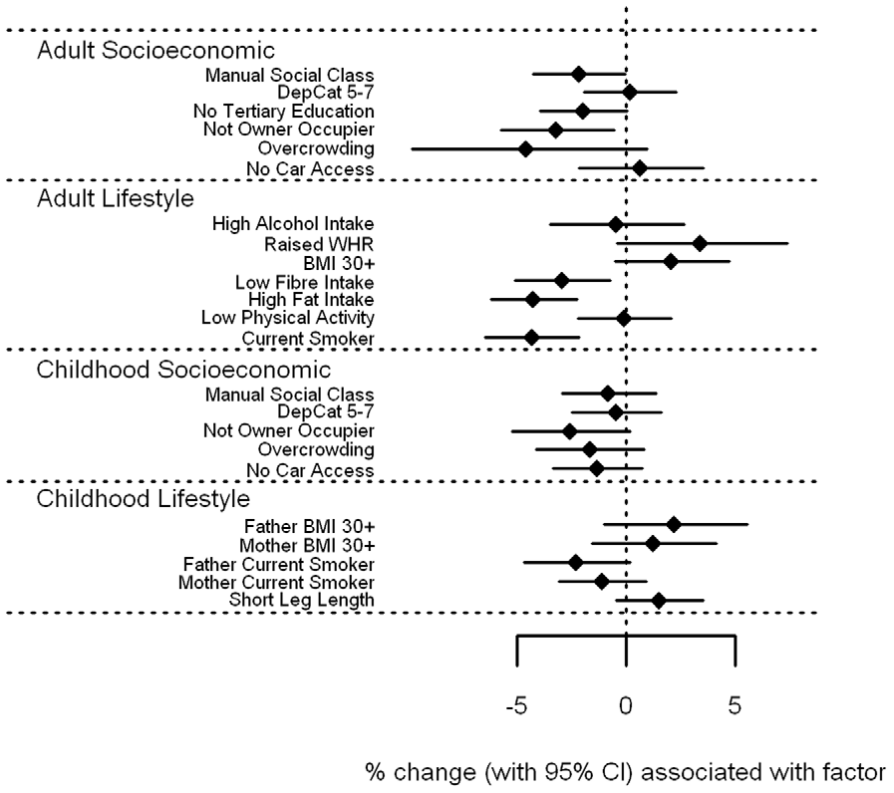
Associations between vitamin E:cholesterol ratio and carotenoids and adult/childhood socioeconomic/lifestyle factors.

For the majority of relevant analyses, results were broadly similar when using categorical measures of lifestyle and deprivation compared to continuous measures, and categorical variables are presented here to present the most complete possible data (continuous data available on request). In comparing dichotomized analyses with continuous analyses, the only trend of note was that maternal BMI generally became a more important determinant of adult offspring vitamin levels in the continuous analyses (except for lycopene and vitamins A and E).

Having shown crude associations of circulating vitamins with markers of deprivation, multivariable models were constructed relating adulthood markers of deprivation to circulating vitamin levels, adjusting for a range of potential confounders, including month, CVD risk factors, physical activity, use of vitamin supplements, and fat and fibre intake (**Table 5**). Vitamin C, α- and β-carotene, leutin, and lycopene all showed generally strong associations with adulthood markers of deprivation in these models. As in the crude unadjusted models, Vitamin A and E had no strong associations with markers of deprivation, even after adjusting for dietary fat and fibre intake.

**Table 5:** Percentage change in vitamin (with 95% CI) by adult socioeconomic deprivation markers.

	Deprivation markers					
	Social Class (I–V)*	DepCat (1–7)*	No tertiary education	Not owner occupier	Overcrowding	No car access
Vitamin C	−5.5% (−7.6, −3.4)*	−3.5% (−5.2, −1.8)*	−12.6% (−17.6, −7.4)*	−25.0% (−30.6, −18.9)*	−27.3% (−38.6, −13.9)*	−20.3% (−26.7, −13.5)*
α-carotene	−6.5% (−9.3, −3.6)*	−2.1% (−4.4, 0.3)	−14.9% (−21.4, −8.0)*	−9.9% (−19.0, 0.3)	−36.7% (−49.6, −20.6)*	−6.5% (−16.4, 4.7)
β-carotene	−6.0% (−8.4, −3.4)*	−4.3% (−6.3, −2.2)*	−9.7% (−15.8, −3.1)†	−17.6% (−25.0, −9.5)*	−27.7% (−40.9, −11.5)†	−4.1% (−13.2, 6.0)
Lutein	−4.7% (−6.2, −3.1)*	−1.9% (−3.1, −0.6)†	−8.0% (−11.8, −4.1)*	−15.7%5 (−20.3, −10.9)*	−17.2% (−26.6, −6.7)†	−7.9% (−13.2, −2.3)†
Lycopene	−3.9% (−6.1, −1.7)†	−2.4% (−4.1, −0.6)†	−8.0% (−13.3, −2.4)†	−19.7% (−25.8, −13.1)*	−2.8% (−18.0, 15.3)	−11.1% (−18.2, −3.3)†
Vitamin A	−0.4% (−1.3, 0.6)	−0.3% (−1.0, 0.5)	−1.0% (−3.5, 1.6)	−0.7% (−4.1, 2.8)	−6.7% (−13.4, 0.4)	−0.7% (−4.3, 3.0)
Vitamin E	−0.5% (−1.4, 0.3)	0.1% (−0.5, 0.8)	−1.6% (−3.8, 0.6)	−0.4% (−3.4, 2.6)	−4.1% (−10.1, 2.2)	4.3% (1.1, 7.7)†

All models adjusted for age, sex, calendar month, cholesterol (logged), HDL cholesterol (logged), SBP, DBP smoking, BMI, physical activity, use of vitamin supplements fat intake and fibre intake.

Social class (I–V) and DepCat (1–7) are expressed as continuous variables.

*indicates p<0.001,

†indicates p<0.01,

‡indicates p<0.05,

No symbol indicates p>0.05.

## Discussion

These findings extend previous work from the British Women's Heart and Health Study [14] to relate a wide range of antioxidant vitamins to a wide range of markers of lifecourse socioeconomic deprivation and adverse lifestyle in a general population. Vitamin C and carotenoids were strongly related to a range of markers of adult socioeconomic circumstance and lifestyle, and to a lesser extent, similar markers from childhood. Associations of these vitamins with adulthood deprivation were independent of conventional CVD risk factors and the limited fat and fibre intake dietary information we had available. Hence, although circulating vitamins might also be surrogate markers of factors such as high fat and low fibre intake (which might themselves increase CVD risk), our results suggest social deprivation remains an important potential confounder in epidemiological studies of blood vitamin levels.

In contrast to vitamin C and the carotenoids, vitamin A and E showed no strong association with markers of adult or childhood socioeconomic position, and this did not markedly change in fully adjusted models. Importantly, we observed a *positive* association of vitamin A and E with some markers of adverse lifestyle. Those who are overweight, or who had an overweight parent were likely to have higher vitamin A and E levels. Speculatively, this may due to the relatively abundant availability of these vitamins even with a high-calorie nutrient-poor diet: vitamin A and E are available from sources such as dairy products and vegetable oil respectively. We did observe some borderline inverse associations of vitamin E with manual social class, not being an owner occupier, and overcrowding, in unadjusted analyses hence our results are not entirely discrepant from the British Women's Heart and Health Study [14], although such associations were broadly absent in our fully adjusted models. Indeed, once vitamin E was ‘corrected’ for by cholesterol levels, its association with adiposity became attenuated, which may support the view that a high-cholesterol diet increases vitamin E as well as circulating cholesterol.

The greater role of current (adult) lifestyle and socioeconomic circumstance compared to that of childhood in determining vitamin levels seems logical: childhood exposure to fruit and vegetables plays an important role in consumption in adulthood [24], but in adulthood current financial and educational circumstances play an important role in vegetable and fruit-associated vitamin intake [25]. Less strong associations of childhood deprivation markers with vitamin levels may be indicative of some degree of social mobility (educational and/or financial) over the lifecourse, which diminishes the association of socioeconomic position in childhood with vitamin intake in adulthood.

The acute effect of alcohol on both serum and hepatic vitamin A metabolism and consequent detectable levels in serum are well known, providing external validity to our data. Our observations are consistent with much previous data showing that acute alcohol administration raises serum levels of vitamin A and does so via a number of pathways relating to retinoid metabolism and mobilisation [26].

Low levels of antioxidant vitamins may be one way in which deprivation causes CVD and other chronic diseases. However, evidence from available RCTs perhaps argues otherwise, including even some data to indicate potential harmful effects of antioxidant vitamin supplementation [11]–[13], [27]. CVD risk scores in the UK are moving towards including deprivation as a risk factor [16], [17], and thus the incremental clinical utility of blood vitamin levels as biomarkers of future risk are also likely to be limited. In addition, it has recently been suggested that if best medical practice was carried out across the socioeconomic spectrum, the difference in CHD rates between the socially advantaged and the less advantaged would almost disappear [28]. Our present work would suggest that such observations apply not only to vitamin C and E, but also to the range of carotenoids and thus we suggest a need for better evidence to support potential causal associations with disease before further costly trials with other antioxidant vitamins are considered.

This cross-sectional study comprises a wide range of antioxidant vitamins and a large trans-generational range of measures of social deprivation and lifestyle, in a cohort designed with a wide range of socioeconomic status, and is powered by a relatively large number of observations in both genders. Data gathered directly from the participant's parents allowed us to make inferences about their childhood circumstances in a manner potentially far less biased by retrospective data gathering. Self-reported information on alcohol consumption, diet and physical activity may be prone to some bias, although such methods are the most pragmatic and clinically utilised for ascertaining such information. The original study was socioeconomically representative of the Paisley/Renfrew region of Renfrewshire, Scotland, but a proportion of ineligible offspring (through age or geographic mobility) may have disturbed this representation, although we anticipate this would probably not substantially impact on the interpretation of our results, especially since the socioeconomic distribution of the population is broadly nationally representative. However, we cannot be sure that cohorts from different regions would display the same associations reported here.

In conclusion, our results emphasise in a striking manner that some circulating levels of antioxidant vitamins are associated with a range of adult and childhood SES measures. These findings should help researchers better contextualize blood antioxidant vitamin levels by illustrating the potential limitations associated with making causal inferences without consideration of social deprivation.

## Supporting Information

File S1 Supplementary tables for Figures 1–8.
(0.39 MB DOC)
