# Correction: Which Circulating Antioxidant Vitamins Are Confounded by Socioeconomic Deprivation? The MIDSPAN Family Study

**DOI:** 10.1371/annotation/530b4aae-2905-45a6-81ee-0c06d0b8ba9b

**Published:** 2010-09-27

**Authors:** Dinesh Talwar, Alex McConnachie, Paul Welsh, Mark Upton, Denis O'Reilly, George Davey Smith, Graham Watt, Naveed Sattar

The legends for Figures 1-8 were published with the words "and carotenoids" inserted into the text by mistake.

