# Supplementary material for: Which Circulating Antioxidant Vitamins Are Confounded by Socioeconomic Deprivation? The MIDSPAN Family Study
Source: PLoS One. 2010 Jun 25;5(6):e11312. doi: 10.1371/journal.pone.0011312 (PMC2892487; doi:10.1371/journal.pone.0011312)
Supplement: File S1 — Supplementary tables for Figures 1–8. (0.39 MB DOC) [file pone.0011312.s001.doc]

**Table s1:** Associations between vitamin C and adult/childhood socioeconomic/lifestyle factors

|  | Effect Estimate (%-change) | 95% CI | p-value |
| --- | --- | --- | --- |
| **Adult Socioeconomic** |  |  |  |
| Manual Social Class | -22.3 | (-26.8, -17.5) | p<0.001 |
| DEPCAT 5-7 | -15.6 | (-20.4, -10.6) | p<0.001 |
| No Tertiary Education | -19.1 | (-23.5, -14.4) | p<0.001 |
| Not Owner Occupier | -35.2 | (-39.7, -30.3) | p<0.001 |
| Overcrowding | -33.0 | (-42.8, -21.6) | p<0.001 |
| No Car Access | -27.6 | (-33.0, -21.7) | p<0.001 |
| **Adult Lifestyle** |  |  |  |
| High Alcohol Intake | -16.5 | (-23.3, -9.1) | p<0.001 |
| Raised WHR | -19.8 | (-27.8, -10.9) | p<0.001 |
| BMI 30+ | -13.4 | (-19.4, -7.0) | p<0.001 |
| Low Fibre Intake | -21.9 | (-26.5, -17.0) | p<0.001 |
| High Fat Intake | -15.9 | (-20.6, -11.0) | p<0.001 |
| Low Physical Activity | -6.3 | (-11.7, -0.5) | p=0.03 |
| Current Smoker | -38.9 | (-42.4, -35.2) | p<0.001 |
| **Childhood Socioeconomic** |  |  |  |
| Manual Social Class | -10.3 | (-15.8, -4.4) | p=0.001 |
| DEPCAT 5-7 | -7.2 | (-12.6, -1.5) | p=0.014 |
| Not Owner Occupier | -14.9 | (-21.5, -7.7) | p<0.001 |
| Overcrowding | -14.9 | (-20.8, -8.6) | p<0.001 |
| No Car Access | -12.0 | (-17.1, -6.7) | p<0.001 |
| **Childhood Lifestyle** |  |  |  |
| Father BMI 30+ | 0.5 | (-8.5, 10.3) | p=0.92 |
| Mother BMI 30+ | 0.8 | (-7.2, 9.6) | p=0.84 |
| Father Current Smoker | 0.7 | (-6.4, 8.3) | p=0.85 |
| Mother Current Smoker | -7.8 | (-13.1, -2.2) | p=0.007 |
| Short Leg Length | -5.9 | (-11.0, -0.5) | p=0.03 |

**Table s2:** Associations between α-carotene and adult/childhood socioeconomic/lifestyle factors

|  | Effect Estimate (%-change) | 95% CI | p-value |
| --- | --- | --- | --- |
| **Adult Socioeconomic** |  |  |  |
| Manual Social Class | -25.2 | (-30.7, -19.3) | p<0.001 |
| DEPCAT 5-7 | -15.6 | (-21.6, -9.1) | p<0.001 |
| No Tertiary Education | -23.8 | (-29.1, -18.1) | p<0.001 |
| Not Owner Occupier | -26.8 | (-33.4, -19.5) | p<0.001 |
| Overcrowding | -38.8 | (-49.9, -25.3) | p<0.001 |
| No Car Access | -22.4 | (-29.8, -14.2) | p<0.001 |
| **Adult Lifestyle** |  |  |  |
| High Alcohol Intake | -31.6 | (-38.7, -23.8) | p<0.001 |
| Raised WHR | -38.3 | (-45.9, -29.5) | p<0.001 |
| BMI 30+ | -32.1 | (-38.0, -25.7) | p<0.001 |
| Low Fibre Intake | -27.1 | (-32.6, -21.1) | p<0.001 |
| High Fat Intake | -16.2 | (-22.1, -9.9) | p<0.001 |
| Low Physical Activity | -11.1 | (-17.6, -4.0) | p=0.003 |
| Current Smoker | -31.8 | (-37.0, -26.3) | p<0.001 |
| **Childhood Socioeconomic** |  |  |  |
| Manual Social Class | -10.5 | (-17.4, -3.0) | p=0.007 |
| DEPCAT 5-7 | -13.0 | (-19.3, -6.2) | p<0.001 |
| Not Owner Occupier | -19.3 | (-27.1, -10.7) | p<0.001 |
| Overcrowding | -16.3 | (-23.6, -8.3) | p<0.001 |
| No Car Access | -9.0 | (-15.6, -1.9) | p=0.01 |
| **Childhood Lifestyle** |  |  |  |
| Father BMI 30+ | -12.5 | (-22.2, -1.5) | p=0.03 |
| Mother BMI 30+ | -11.5 | (-20.3, -1.8) | p=0.02 |
| Father Current Smoker | -5.1 | (-13.4, 4.0) | p=0.26 |
| Mother Current Smoker | -7.5 | (-14.1, -0.3) | p=0.04 |
| Short Leg Length | -5.7 | (-12.2, 1.2) | p=0.10 |

**Table s3:** Associations between β-carotene and adult/childhood socioeconomic/lifestyle factors

|  | Effect Estimate (%-change) | 95% CI | p-value |
| --- | --- | --- | --- |
| **Adult Socioeconomic** |  |  |  |
| Manual Social Class | -21.2 | (-26.5, -15.4) | p<0.001 |
| DEPCAT 5-7 | -16.0 | (-21.5, -10.0) | p<0.001 |
| No Tertiary Education | -20.6 | (-25.7, -15.1) | p<0.001 |
| Not Owner Occupier | -29.4 | (-35.3, -23.0) | p<0.001 |
| Overcrowding | -32.7 | (-43.9, -19.1) | p<0.001 |
| No Car Access | -17.6 | (-24.9, -9.7) | p<0.001 |
| **Adult Lifestyle** |  |  |  |
| High Alcohol Intake | -44.6 | (-49.8, -38.9) | p<0.001 |
| Raised WHR | -29.7 | (-37.8, -20.5) | p<0.001 |
| BMI 30+ | -24.5 | (-30.5, -17.9) | p<0.001 |
| Low Fibre Intake | -27.3 | (-32.3, -21.9) | p<0.001 |
| High Fat Intake | -12.0 | (-17.7, -5.9) | p<0.001 |
| Low Physical Activity | -11.8 | (-17.8, -5.3) | p=0.001 |
| Current Smoker | -29.2 | (-34.1, -23.9) | p<0.001 |
| **Childhood Socioeconomic** |  |  |  |
| Manual Social Class | -12.3 | (-18.6, -5.5) | p=0.001 |
| DEPCAT 5-7 | -10.0 | (-16.1, -3.4) | p=0.003 |
| Not Owner Occupier | -18.2 | (-25.5, -10.1) | p<0.001 |
| Overcrowding | -16.7 | (-23.4, -9.4) | p<0.001 |
| No Car Access | -8.8 | (-15.0, -2.3) | p=0.01 |
| **Childhood Lifestyle** |  |  |  |
| Father BMI 30+ | -4.7 | (-14.6, 6.4) | p=0.39 |
| Mother BMI 30+ | -5.7 | (-14.4, 3.9) | p=0.23 |
| Father Current Smoker | -3.6 | (-11.4, 5.0) | p=0.41 |
| Mother Current Smoker | -9.2 | (-15.3, -2.6) | p=0.007 |
| Short Leg Length | -7.3 | (-13.2, -1.0) | p=0.02 |

**Table s4:** Associations between lutein and adult/childhood socioeconomic/lifestyle factors

|  | Effect Estimate (%-change) | 95% CI | p-value |
| --- | --- | --- | --- |
| **Adult Socioeconomic** |  |  |  |
| Manual Social Class | -17.4 | (-20.6, -14.0) | p<0.001 |
| DEPCAT 5-7 | -8.6 | (-12.1, -4.9) | p<0.001 |
| No Tertiary Education | -13.5 | (-16.8, -10.2) | p<0.001 |
| Not Owner Occupier | -22.3 | (-26.0, -18.3) | p<0.001 |
| Overcrowding | -20.0 | (-28.0, -11.2) | p<0.001 |
| No Car Access | -15.4 | (-19.8, -10.8) | p<0.001 |
| **Adult Lifestyle** |  |  |  |
| High Alcohol Intake | -14.8 | (-19.5, -9.7) | p<0.001 |
| Raised WHR | -15.0 | (-20.8, -8.8) | p<0.001 |
| BMI 30+ | -11.5 | (-15.7, -7.1) | p<0.001 |
| Low Fibre Intake | -11.4 | (-15.0, -7.6) | p<0.001 |
| High Fat Intake | -9.9 | (-13.4, -6.4) | p<0.001 |
| Low Physical Activity | -3.0 | (-6.9, 1.0) | p=0.13 |
| Current Smoker | -18.9 | (-22.2, -15.5) | p<0.001 |
| **Childhood Socioeconomic** |  |  |  |
| Manual Social Class | -10.0 | (-13.8, -6.0) | p<0.001 |
| DEPCAT 5-7 | -6.7 | (-10.4, -2.8) | p=0.001 |
| Not Owner Occupier | -12.0 | (-16.7, -7.1) | p<0.001 |
| Overcrowding | -10.4 | (-14.7, -5.9) | p<0.001 |
| No Car Access | -7.9 | (-11.5, -4.1) | p<0.001 |
| **Childhood Lifestyle** |  |  |  |
| Father BMI 30+ | 1.2 | (-5.1, 7.9) | p=0.71 |
| Mother BMI 30+ | -8.4 | (-13.4, -3.1) | p=0.002 |
| Father Current Smoker | -1.7 | (-6.4, 3.4) | p=0.51 |
| Mother Current Smoker | -2.4 | (-6.3, 1.6) | p=0.24 |
| Short Leg Length | -0.9 | (-4.5, 3.0) | p=0.66 |

**Table s5:** Associations between lycopene and adult/childhood socioeconomic/lifestyle factors

|  | Effect Estimate (%-change) | 95% CI | p-value |
| --- | --- | --- | --- |
| **Adult Socioeconomic** |  |  |  |
| Manual Social Class | -13.5 | (-18.3, -8.4) | p<0.001 |
| DEPCAT 5-7 | -7.5 | (-12.5, -2.2) | p=0.006 |
| No Tertiary Education | -12.3 | (-16.9, -7.5) | p<0.001 |
| Not Owner Occupier | -24.3 | (-29.5, -18.8) | p<0.001 |
| Overcrowding | -19.1 | (-30.3, -6.0) | p=0.006 |
| No Car Access | -19.8 | (-25.6, -13.6) | p<0.001 |
| **Adult Lifestyle** |  |  |  |
| High Alcohol Intake | -0.1 | (-8.0, 8.5) | p=0.99 |
| Raised WHR | -17.9 | (-25.7, -9.3) | p<0.001 |
| BMI 30+ | -14.3 | (-20.0, -8.3) | p<0.001 |
| Low Fibre Intake | -6.3 | (-11.7, -0.5) | p=0.03 |
| High Fat Intake | -8.9 | (-13.7, -3.8) | p=0.001 |
| Low Physical Activity | -4.5 | (-9.8, 1.1) | p=0.12 |
| Current Smoker | -12.4 | (-17.5, -7.0) | p<0.001 |
| **Childhood Socioeconomic** |  |  |  |
| Manual Social Class | -3.3 | (-8.9, 2.6) | p=0.27 |
| DEPCAT 5-7 | -3.2 | (-8.5, 2.3) | p=0.25 |
| Not Owner Occupier | -3.4 | (-10.5, 4.2) | p=0.37 |
| Overcrowding | -7.0 | (-13.1, -0.5) | p=0.04 |
| No Car Access | -6.7 | (-11.8, -1.4) | p=0.02 |
| **Childhood Lifestyle** |  |  |  |
| Father BMI 30+ | 2.4 | (-6.1, 11.8) | p=0.59 |
| Mother BMI 30+ | 0.6 | (-6.9, 8.6) | p=0.89 |
| Father Current Smoker | 7.9 | (0.8, 15.4) | p=0.03 |
| Mother Current Smoker | 1.3 | (-4.2, 7.0) | p=0.66 |
| Short Leg Length | -2.0 | (-7.1, 3.4) | p=0.46 |

**Table s6:** Associations between α-carotene/Cholesterol ratio and adult/childhood socioeconomic/lifestyle factors

|  | Effect Estimate (%-change) | 95% CI | p-value |
| --- | --- | --- | --- |
| **Adult Socioeconomic** |  |  |  |
| Manual Social Class | -25.2 | (-30.7, -19.3) | p<0.001 |
| DEPCAT 5-7 | -15.6 | (-21.6, -9.1) | p<0.001 |
| No Tertiary Education | -23.8 | (-29.1, -18.1) | p<0.001 |
| Not Owner Occupier | -26.8 | (-33.4, -19.5) | p<0.001 |
| Overcrowding | -38.8 | (-49.9, -25.3) | p<0.001 |
| No Car Access | -22.4 | (-29.8, -14.2) | p<0.001 |
| **Adult Lifestyle** |  |  |  |
| High Alcohol Intake | -31.6 | (-38.7, -23.8) | p<0.001 |
| Raised WHR | -38.3 | (-45.9, -29.5) | p<0.001 |
| BMI 30+ | -32.1 | (-38.0, -25.7) | p<0.001 |
| Low Fibre Intake | -27.1 | (-32.6, -21.1) | p<0.001 |
| High Fat Intake | -16.2 | (-22.1, -9.9) | p<0.001 |
| Low Physical Activity | -11.1 | (-17.6, -4.0) | p=0.003 |
| Current Smoker | -31.8 | (-37.0, -26.3) | p<0.001 |
| **Childhood Socioeconomic** |  |  |  |
| Manual Social Class | -10.5 | (-17.4, -3.0) | p=0.007 |
| DEPCAT 5-7 | -13.0 | (-19.3, -6.2) | p<0.001 |
| Not Owner Occupier | -19.3 | (-27.1, -10.7) | p<0.001 |
| Overcrowding | -16.3 | (-23.6, -8.3) | p<0.001 |
| No Car Access | -9.0 | (-15.6, -1.9) | p=0.01 |
| **Childhood Lifestyle** |  |  |  |
| Father BMI 30+ | -12.5 | (-22.2, -1.5) | p=0.03 |
| Mother BMI 30+ | -11.5 | (-20.3, -1.8) | p=0.02 |
| Father Current Smoker | -5.1 | (-13.4, 4.0) | p=0.26 |
| Mother Current Smoker | -7.5 | (-14.1, -0.3) | p=0.04 |
| Short Leg Length | -5.7 | (-12.2, 1.2) | p=0.10 |

**Table s7:** Associations between β-carotene/cholesterol ratio and adult/childhood socioeconomic/lifestyle factors

|  | Effect Estimate (%-change) | 95% CI | p-value |
| --- | --- | --- | --- |
| **Adult Socioeconomic** |  |  |  |
| Manual Social Class | -21.2 | (-26.5, -15.4) | p<0.001 |
| DEPCAT 5-7 | -16.0 | (-21.5, -10.0) | p<0.001 |
| No Tertiary Education | -20.6 | (-25.7, -15.1) | p<0.001 |
| Not Owner Occupier | -29.4 | (-35.3, -23.0) | p<0.001 |
| Overcrowding | -32.7 | (-43.9, -19.1) | p<0.001 |
| No Car Access | -17.6 | (-24.9, -9.7) | p<0.001 |
| **Adult Lifestyle** |  |  |  |
| High Alcohol Intake | -44.6 | (-49.8, -38.9) | p<0.001 |
| Raised WHR | -29.7 | (-37.8, -20.5) | p<0.001 |
| BMI 30+ | -24.5 | (-30.5, -17.9) | p<0.001 |
| Low Fibre Intake | -27.3 | (-32.3, -21.9) | p<0.001 |
| High Fat Intake | -12.0 | (-17.7, -5.9) | p<0.001 |
| Low Physical Activity | -11.8 | (-17.8, -5.3) | p=0.001 |
| Current Smoker | -29.2 | (-34.1, -23.9) | p<0.001 |
| **Childhood Socioeconomic** |  |  |  |
| Manual Social Class | -12.3 | (-18.6, -5.5) | p=0.001 |
| DEPCAT 5-7 | -10.0 | (-16.1, -3.4) | p=0.003 |
| Not Owner Occupier | -18.2 | (-25.5, -10.1) | p<0.001 |
| Overcrowding | -16.7 | (-23.4, -9.4) | p<0.001 |
| No Car Access | -8.8 | (-15.0, -2.3) | p=0.01 |
| **Childhood Lifestyle** |  |  |  |
| Father BMI 30+ | -4.7 | (-14.6, 6.4) | p=0.39 |
| Mother BMI 30+ | -5.7 | (-14.4, 3.9) | p=0.23 |
| Father Current Smoker | -3.6 | (-11.4, 5.0) | p=0.41 |
| Mother Current Smoker | -9.2 | (-15.3, -2.6) | p=0.007 |
| Short Leg Length | -7.3 | (-13.2, -1.0) | p=0.02 |

**Table s8:** Associations between lutein/cholesterol ratio and adult/childhood socioeconomic/lifestyle factors

|  | Effect Estimate (%-change) | 95% CI | p-value |
| --- | --- | --- | --- |
| **Adult Socioeconomic** |  |  |  |
| Manual Social Class | -17.4 | (-20.6, -14.0) | p<0.001 |
| DEPCAT 5-7 | -8.6 | (-12.1, -4.9) | p<0.001 |
| No Tertiary Education | -13.5 | (-16.8, -10.2) | p<0.001 |
| Not Owner Occupier | -22.3 | (-26.0, -18.3) | p<0.001 |
| Overcrowding | -20.0 | (-28.0, -11.2) | p<0.001 |
| No Car Access | -15.4 | (-19.8, -10.8) | p<0.001 |
| **Adult Lifestyle** |  |  |  |
| High Alcohol Intake | -14.8 | (-19.5, -9.7) | p<0.001 |
| Raised WHR | -15.0 | (-20.8, -8.8) | p<0.001 |
| BMI 30+ | -11.5 | (-15.7, -7.1) | p<0.001 |
| Low Fibre Intake | -11.4 | (-15.0, -7.6) | p<0.001 |
| High Fat Intake | -9.9 | (-13.4, -6.4) | p<0.001 |
| Low Physical Activity | -3.0 | (-6.9, 1.0) | p=0.13 |
| Current Smoker | -18.9 | (-22.2, -15.5) | p<0.001 |
| **Childhood Socioeconomic** |  |  |  |
| Manual Social Class | -10.0 | (-13.8, -6.0) | p<0.001 |
| DEPCAT 5-7 | -6.7 | (-10.4, -2.8) | p=0.001 |
| Not Owner Occupier | -12.0 | (-16.7, -7.1) | p<0.001 |
| Overcrowding | -10.4 | (-14.7, -5.9) | p<0.001 |
| No Car Access | -7.9 | (-11.5, -4.1) | p<0.001 |
| **Childhood Lifestyle** |  |  |  |
| Father BMI 30+ | 1.2 | (-5.1, 7.9) | p=0.71 |
| Mother BMI 30+ | -8.4 | (-13.4, -3.1) | p=0.002 |
| Father Current Smoker | -1.7 | (-6.4, 3.4) | p=0.51 |
| Mother Current Smoker | -2.4 | (-6.3, 1.6) | p=0.24 |
| Short Leg Length | -0.9 | (-4.5, 3.0) | p=0.66 |

**Table s9:** Associations between lycopene/cholesterol ratio and adult/childhood socioeconomic/lifestyle factors

|  | Effect Estimate (%-change) | 95% CI | p-value |
| --- | --- | --- | --- |
| **Adult Socioeconomic** |  |  |  |
| Manual Social Class | -13.5 | (-18.3, -8.4) | p<0.001 |
| DEPCAT 5-7 | -7.5 | (-12.5, -2.2) | p=0.006 |
| No Tertiary Education | -12.3 | (-16.9, -7.5) | p<0.001 |
| Not Owner Occupier | -24.3 | (-29.5, -18.8) | p<0.001 |
| Overcrowding | -19.1 | (-30.3, -6.0) | p=0.006 |
| No Car Access | -19.8 | (-25.6, -13.6) | p<0.001 |
| **Adult Lifestyle** |  |  |  |
| High Alcohol Intake | -0.1 | (-8.0, 8.5) | p=0.99 |
| Raised WHR | -17.9 | (-25.7, -9.3) | p<0.001 |
| BMI 30+ | -14.3 | (-20.0, -8.3) | p<0.001 |
| Low Fibre Intake | -6.3 | (-11.7, -0.5) | p=0.03 |
| High Fat Intake | -8.9 | (-13.7, -3.8) | p=0.001 |
| Low Physical Activity | -4.5 | (-9.8, 1.1) | p=0.12 |
| Current Smoker | -12.4 | (-17.5, -7.0) | p<0.001 |
| **Childhood Socioeconomic** |  |  |  |
| Manual Social Class | -3.3 | (-8.9, 2.6) | p=0.27 |
| DEPCAT 5-7 | -3.2 | (-8.5, 2.3) | p=0.25 |
| Not Owner Occupier | -3.4 | (-10.5, 4.2) | p=0.37 |
| Overcrowding | -7.0 | (-13.1, -0.5) | p=0.04 |
| No Car Access | -6.7 | (-11.8, -1.4) | p=0.02 |
| **Childhood Lifestyle** |  |  |  |
| Father BMI 30+ | 2.4 | (-6.1, 11.8) | p=0.59 |
| Mother BMI 30+ | 0.6 | (-6.9, 8.6) | p=0.89 |
| Father Current Smoker | 7.9 | (0.8, 15.4) | p=0.03 |
| Mother Current Smoker | 1.3 | (-4.2, 7.0) | p=0.66 |
| Short Leg Length | -2.0 | (-7.1, 3.4) | p=0.46 |

**Table s10:** Associations between vitamin A and adult/childhood socioeconomic/lifestyle factors

|  | Effect Estimate (%-change) | 95% CI | p-value |
| --- | --- | --- | --- |
| **Adult Socioeconomic** |  |  |  |
| Manual Social Class | -0.2 | (-2.7, 2.3) | p=0.86 |
| DEPCAT 5-7 | 2.0 | (-0.5, 4.4) | p=0.12 |
| No Tertiary Education | 0.5 | (-1.8, 2.9) | p=0.66 |
| Not Owner Occupier | -0.1 | (-3.2, 3.0) | p=0.93 |
| Overcrowding | -6.1 | (-12.0, 0.2) | p=0.06 |
| No Car Access | 0.4 | (-2.8, 3.7) | p=0.81 |
| **Adult Lifestyle** |  |  |  |
| High Alcohol Intake | 9.5 | (5.7, 13.5) | p<0.001 |
| Raised WHR | 4.6 | (0.2, 9.3) | p=0.04 |
| BMI 30+ | 1.6 | (-1.4, 4.7) | p=0.29 |
| Low Fibre Intake | 1.3 | (-1.2, 4.0) | p=0.32 |
| High Fat Intake | 2.4 | (0.0, 4.9) | p=0.05 |
| Low Physical Activity | 1.1 | (-1.4, 3.6) | p=0.40 |
| Current Smoker | 2.9 | (0.2, 5.6) | p=0.03 |
| **Childhood Socioeconomic** |  |  |  |
| Manual Social Class | -2.5 | (-5.0, 0.0) | p=0.05 |
| DEPCAT 5-7 | -0.4 | (-2.8, 2.1) | p=0.76 |
| Not Owner Occupier | -2.2 | (-5.4, 1.0) | p=0.18 |
| Overcrowding | -1.6 | (-4.5, 1.3) | p=0.28 |
| No Car Access | -1.2 | (-3.5, 1.2) | p=0.34 |
| **Childhood Lifestyle** |  |  |  |
| Father BMI 30+ | 7.2 | (3.3, 11.3) | p<0.001 |
| Mother BMI 30+ | 5.7 | (2.3, 9.3) | p=0.001 |
| Father Current Smoker | 1.9 | (-1.0, 5.0) | p=0.20 |
| Mother Current Smoker | 0.2 | (-2.2, 2.7) | p=0.86 |
| Short Leg Length | 0.3 | (-2.0, 2.6) | p=0.80 |

**Table s11:** Associations between vitamin E and adult/childhood socioeconomic/lifestyle factors

|  | Effect Estimate (%-change) | 95% CI | p-value |
| --- | --- | --- | --- |
| **Adult Socioeconomic** |  |  |  |
| Manual Social Class | -2.7 | (-5.1, -0.2) | p=0.04 |
| DEPCAT 5-7 | 0.0 | (-2.4, 2.5) | p=0.98 |
| No Tertiary Education | -2.0 | (-4.3, 0.4) | p=0.10 |
| Not Owner Occupier | -3.2 | (-6.2, -0.1) | p=0.04 |
| Overcrowding | -5.9 | (-11.9, 0.6) | p=0.07 |
| No Car Access | 1.0 | (-2.3, 4.4) | p=0.57 |
| **Adult Lifestyle** |  |  |  |
| High Alcohol Intake | 2.2 | (-1.4, 6.0) | p=0.23 |
| Raised WHR | 9.5 | (4.8, 14.4) | p<0.001 |
| BMI 30+ | 6.6 | (3.5, 9.9) | p<0.001 |
| Low Fibre Intake | -0.7 | (-3.3, 1.9) | p=0.58 |
| High Fat Intake | -1.5 | (-3.9, 0.9) | p=0.22 |
| Low Physical Activity | 1.6 | (-0.9, 4.2) | p=0.20 |
| Current Smoker | -2.7 | (-5.2, -0.1) | p=0.04 |
| **Childhood Socioeconomic** |  |  |  |
| Manual Social Class | -2.3 | (-4.9, 0.3) | p=0.08 |
| DEPCAT 5-7 | 0.6 | (-1.9, 3.1) | p=0.63 |
| Not Owner Occupier | -3.1 | (-6.3, 0.2) | p=0.06 |
| Overcrowding | -2.4 | (-5.3, 0.6) | p=0.12 |
| No Car Access | -1.1 | (-3.6, 1.3) | p=0.36 |
| **Childhood Lifestyle** |  |  |  |
| Father BMI 30+ | 4.4 | (0.4, 8.5) | p=0.03 |
| Mother BMI 30+ | 1.1 | (-2.3, 4.6) | p=0.52 |
| Father Current Smoker | -1.4 | (-4.4, 1.6) | p=0.34 |
| Mother Current Smoker | -0.3 | (-2.8, 2.2) | p=0.79 |
| Short Leg Length | 4.8 | (2.4, 7.3) | p<0.001 |

**Table s12:** Associations between vitamin E/cholesterol ratio and adult/childhood socioeconomic/lifestyle factors

|  | Effect Estimate (%-change) | 95% CI | p-value |
| --- | --- | --- | --- |
| **Adult Socioeconomic** |  |  |  |
| Manual Social Class | -2.2 | (-4.2, 0.0) | p=0.05 |
| DEPCAT 5-7 | 0.2 | (-1.9, 2.3) | p=0.88 |
| No Tertiary Education | -2.0 | (-3.9, 0.1) | p=0.06 |
| Not Owner Occupier | -3.2 | (-5.7, -0.6) | p=0.02 |
| Overcrowding | -4.6 | (-9.8, 0.9) | p=0.10 |
| No Car Access | 0.6 | (-2.1, 3.5) | p=0.65 |
| **Adult Lifestyle** |  |  |  |
| High Alcohol Intake | -0.5 | (-3.5, 2.6) | p=0.76 |
| Raised WHR | 3.4 | (-0.4, 7.3) | p=0.08 |
| BMI 30+ | 2.1 | (-0.5, 4.7) | p=0.12 |
| Low Fibre Intake | -2.9 | (-5.1, -0.7) | p=0.01 |
| High Fat Intake | -4.2 | (-6.2, -2.3) | p<0.001 |
| Low Physical Activity | -0.1 | (-2.2, 2.1) | p=0.93 |
| Current Smoker | -4.3 | (-6.4, -2.2) | p<0.001 |
| **Childhood Socioeconomic** |  |  |  |
| Manual Social Class | -0.8 | (-2.9, 1.4) | p=0.47 |
| DEPCAT 5-7 | -0.5 | (-2.5, 1.6) | p=0.66 |
| Not Owner Occupier | -2.6 | (-5.2, 0.2) | p=0.07 |
| Overcrowding | -1.7 | (-4.1, 0.8) | p=0.19 |
| No Car Access | -1.3 | (-3.3, 0.7) | p=0.20 |
| **Childhood Lifestyle** |  |  |  |
| Father BMI 30+ | 2.2 | (-1.0, 5.5) | p=0.18 |
| Mother BMI 30+ | 1.2 | (-1.6, 4.1) | p=0.39 |
| Father Current Smoker | -2.3 | (-4.7, 0.2) | p=0.07 |
| Mother Current Smoker | -1.1 | (-3.1, 0.9) | p=0.28 |
| Short Leg Length | 1.5 | (-0.5, 3.5) | p=0.14 |
